# Protein phosphatase 2A activation mechanism contributes to JS-K induced caspase-dependent apoptosis in human hepatocellular carcinoma cells

**DOI:** 10.1186/s13046-018-0823-2

**Published:** 2018-07-09

**Authors:** Ling Liu, Zile Huang, Jingjing Chen, Jiangang Wang, Shuying Wang

**Affiliations:** Department of Pharmacy, Henan University of Science and Technology, 263 Kaiyuan Avenue, Luoyang, 471023 China

**Keywords:** Hepatocellular carcinoma, Nitric oxide, JS-K, Protein phosphatase 2A, Apoptosis

## Abstract

**Background:**

JS-K is a nitric oxide (NO) donor and could generate intracellularly high levels of NO. The study explores PP2A as a tumor suppressor is a major determinant mediating JS-K-caused apoptosis in human hepatocellular carcinoma (HCC) cells.

**Methods:**

The human HCC cell lines (PLC5, Huh-7, Bel-7402, SMMC-7721 and HepG2) were used to assess effects of JS-K on cell viability, apoptosis induction and PP2A activation. Effects of JS-K on cell morphology, mitochondrial membrane potential, apoptosis and NO levels were determined in HCC cells expressing PP2A. Simultaneously, the expression of PP2A family including PP2A-A(α/β), PP2A-B55, PP2A-C(α/β) and the substrates of PP2A, such as β-catenin, c-Myc and p-Bcl-2 (Ser70) were detected in sensitive HCC cells. Furthermore, the role of NO in mediating the expression of PP2A was further validated with Z-VAD-FMK (a caspase inhibitor), Carboxy-PTIO (a NO scavenger), okadaic acid (OA, a PP2A inhibitor) and FTY720 (a PP2A agonist) in JS-K treated cells. In addition, the genetic manuplation of PP2A including overexpression and knockdown have been also performed in JS-K treated cells. Moreover, the rat model of primary hepatic carcinoma was established with diethylnitrosamine for 16 weeks to verify the anti-tumor effects of JS-K in vivo. Immunohistochemical and Western blot analysis were used to determine the expression of proteins in rat primary hepatic carcinoma tissues.

**Results:**

JS-K significantly inhibited cell proliferation, increased apoptosis rate and activated PP2A activity in five HCC cells viability, especially SMMC7721 and HepG2 cells. It was characterized by loss of mitochondrial membrane potential, significant externalization of phosphatidylserine, nuclear morphological changes. Moreover, JS-K enhanced Bax-to-Bcl-2 ratio, released cytochrome c (Cyt c) from mitochondria, activated cleaved-caspase-9/3 and the cleavage of PARP, and decreased the expression of X-linked inhibitor of apoptosis protein (XIAP). Both Z-VAD-FMK and Carboxy-PTIO suppressed the activation of cleaved-caspase-9/3 and of cleaved-PARP in JS-K-treated sensitive HCC cells. Simultaneously, JS-K treatment could lead to the activation of protein phosphatase 2A-C (PP2A-C) but not PP2A-A and PP2A-B55, which subsequently inactivated and dephosphorylated the PP2A substrates including β-catenin, c-Myc, and p-Bcl-2 (Ser70). However, silencing PP2A-C could abolish both the activation of PP2A-C and down-regulation of β-catenin, c-Myc and p-Bcl-2 (Ser70) in sensitive HCC cells. Conversely, PP2A overexpression could enhance the effects of JS-K on activation of PP2A and down-regulation of β-catenin, c-Myc and p-Bcl-2 (Ser70). In addition, adding okadaic acid (OA), a PP2A inhibitor, abolished the effects of JS-K on apoptosis induction, PP2A activation and the substrates of PP2A dephosphorylation; FTY720, a PP2A agonist, enhanced the effects of JS-K including apoptosis induction, PP2A activation and the substrates of PP2A dephosphorylation. The mice exhibited a lower number and smaller tumor nodules in response to JS-K-treated group. A marked increase in the number of hepatocytes with PCNA-positive nuclei (proliferating cells) was evident in DEN group and tended to decrease with JS-K treatment. Furthermore, JS-K treatment could induce PP2A activation and the substrates of PP2A inactivation such as β-catenin, c-Myc and p-Bcl-2(Ser70) in DEN-induced hepatocarcinogenesis.

**Conclusions:**

High levels of NO released from JS-K induces a caspase-dependent apoptosis through PP2A activation.

## Background

Protein phosphatase 2A (PP2A) is a member of phosphoprotein phosphatase (PPP) family which comprises cellular serine/threonine phosphatases [1–3]. Actually, decreased activity of PP2A has been reported as a recurrent alteration in many types of cancer [4]. Moreover, several cellular inhibitors of PP2A have been identified in a variety of cancer types [3, 5]. CIP2A as a PP2A inhibitor is overexpressed in many human malignancies [3]. However, FTY720 as a PP2A activator could possess potent antitumor properties via restoration of PP2A activity [6]. Ceramides as another PP2A activator belong to structural components of the cell membrane, which have potent signaling properties that result in cell apoptosis, senescence, or cell-cycle arrest [7–9]. In addition, PP2A as a tumor suppressor negatively regulates many proliferative signaling pathways associated with cancer progression by dephosphorylating crucial proteins in these pathways such as Wnt/β-catenin, PI3K/Akt and ERK/ MAPK signaling pathway [4, 10, 11].

Nitric oxide (NO), a major signaling molecule, is involved in various physiological and pathological processes. High level of NO has the cytotoxic and apoptosis-inducing effects on oncogenesis. NO is often derived from both the endogenous way by stimulating NO syntheses and the exogenous way through NO donor [12]. O^2^-(2,4-dinitrophenyl) 1-[(4-ethoxycarbonyl)piperazin-1-yl]diazen-1-ium-1,2-diolate (JS-K,C_13_H_16_N_6_O_8_) is a diazeniumdiolate-based NO donor and is highly cytotoxic to several types of human cancer cells, such as acute lymphoblastic leukemia [13], hepatocellular carcinoma [14], prostate cancer cells [15] or murine erythroleukemia cells [16]. Moreover, JS-K as a lead NO donor selectively exhibits antitumor effects towards cancer cells while has no significant toxicity toward normal cells [17]. The nonobese diabetic-severe combined immune deficient mice intravenously injected with JS-K had not display significant hypotension [18]. Simultaneously, JS-K also inhibited the growth and induced apoptosis of tumor cell lines through different signaling pathway. Ren [19] demonstrated that JS-K inhibited Hep 3B hepatoma cell growth and induced c-Jun phosphorylation via multiple MAP kinase pathways. JS-K also has been shown to inhibit the prostate cancer cells growth, which could be attributed to inhibit WNT- and AR-signaling via NO-release [20]. Furthermore, NO also induced cell apoptosis through increasing the level of ceramides [21], which could increase the activity of PP2A.

Therefore, some signaling pathways such as Wnt/β-catenin, MAPK and Bcl-2 family are meeting points between JS-K and PP2A. In our previous study, JS-K inhibited the proliferation of HepG2 cells and significantly induced apoptosis via Ca^2+^/caspase-3-mediated pathway [22]. The present study demonstrated that the anti-proliferative and apoptosis- inductive effects of JS-K were attributed to increase the activity of PP2A, which caused dephosphorylation of its downstream responsive substrates like β-catenin, c-Myc, and Bcl-2. Furthermore, JS-K increased Bax-to-Bcl-2 ratio, released Cyt c from mitochondria and increased the activities of cleaved-caspase-9/3.Therefore, PP2A activation mechanism contributes to JS-K induced caspase-dependent apoptosis in human hepatocellular carcinoma cells.

## Methods

### Reagents and antibodies

JS-K, PP2A-Cα (siRNA) and control siRNA-A, antibody against to PP2A-A (α/β), PP2A-B55, PP2A-C (α/β) and p-Bcl-2(Ser70) were purchased from Santa Cruz Biotechnology (San Diego, CA). JS-K was dissolved in 100% DMSO to a concentration of 10 mM as a stock solution. The final concentration of DMSO did not exceed 0.1% throughout the study. Reagents used in the present study included cell counting kit-8(CCK-8) (Dojindo Laboratories, Kumamoto, Japan), Annexin V-FITC/PI kit (BD Biosciences, NJ, San Diego, CA, USA), DAPI staining solution, 3-Amino, 4-aminomethyl-2′,7′-difluorescein, diacetate (DAF-FM DA), 5,5′,6,6′-tetrachloro-1,1′,3,3′- tetraethylbenzimidazolcarbocyanine iodide (JC-1), Carboxy-PTIO, cell lysis buffer kit for Western, enhanced BCA Protein Assay Kit (Beyotime, Haimen, China). Human Protein phosphatase-2A (PP2A) Elisa kit was supplied with Jianglai Institute of Biotechnology (Shanghai, China). Okadaic acid (OA), Z-VAD (OMe)-FMK and antibodies for Bax, Bcl-2, cytochrome c, pro-caspase-3, cleaved-caspase-3, pro-caspase-9, cleaved caspase-9, PARP, cleaved PARP, XIAP, β-catenin, c-Myc, β-actin and COX IV were supplied from Cell Signaling Technology (Beverly, MA, USA). HRP-conjugated affinipure goat anti-mouse IgG and HRP-conjugated affinipure goat anti-rabbit IgG were supplied from Proteintech Group, Inc. (Wuhan, China). FTY720 was purchased from Sigma-Aldrich Chemical Company (St. Louis, MO, USA). PPP2CA cDNA (PP2A-Cα, GV230 carrier, NheI/AgeI enzymatic cutting) was purchased from Shanghai Genechem Co.,Ltd.

### Cells culture

The human hepatocellular carcinoma cells (HCC) (PLC5, Huh-7, Bel-7402, SMMC-7721 and HepG2) were purchased from Shanghai Institute of Cell Biology (Shanghai, China). PLC5, Bel-7402, and SMMC-7721 cells were maintained in RPMI 1640 medium (Gibco, Invitrogen) while Huh-7 and HepG2 cells were cultured in high glucose DMEM (Gibco, Invitrogen). Both medium were supplemented with 10% FBS. Penicillin 100 U/mL and streptomycin 100 μg/mL were added to the cultured medium. All cells were cultured at 37 °C in a humidified atmosphere containing 5% CO_2_.

### Animals

A total of 32 eight-week-old male Wistar rats, weighing 180 ± 10 g, were acquired from Experimental Animal Center of Medical College in Henan University of Science and Technology (Luoyang, China). Animals were acclimatized under standardized conditions (23 ± 2 °C, 60 ± 10% humidity, 12 h light/dark cycle) for one week prior to use. All experimental procedures were approved by and performed in accordance with the guidelines set out by the Institutional Animal Experiment Committee of Henan University of Science and Technology, China.

### Cell counting Kti-8 (CCK-8) proliferation assays

The cells at a final density of 1 × 10^4^ cells/well were seeded into 96-well cell plates overnight and incubated with various concentrations of JS-K for 24, 48 or 72 h. Thereafter, the medium with JS-K was removed and rapidly replaced with 100 μL medium containing 10 μL CCK-8 reagent. After incubation at 37 °C for 2 h, the absorbance was measured using a spectrophotometer (Tecan, Switzerland) at a wavelength of 450 nm. Experiments were conducted in triplicate. Inhibition rate (%) = [(OD _Control_ –OD _Treated_)/ OD _Control_] × 100%.

### DAPI staining and crystal violet staining

HCC cells (5 × 10^5^cells) were seeded into 6-well cell plate for 12 h and then treated with JS-K for 24 h. The typical morphology of cells was confirmed by phase contrast microscopy. Then the cells were washed with PBS twice, and incubated with DAPI in accordance with the manufacturer’s instructions. After staining, the cells were immediately observed by a fluorescence microscope (Olympus, IX-70, Japan). In addition, the cells were fixed with 4% paraformaldehyde and stained with 0.5% crystal violet while they were exposed to JS-K for 24 h and observed by phase contrast microscopy.

### Apoptosis detection

The cells were treated with different concentrations of JS-K for 24 h. In addition, cells were pre-treated with indicated concentration of Carboxy-PTIO (50 μM), OA (1 nM) or FTY720 (2.5 μM) for 1 h, and then the cells were treated with JS-K for indicated time. Thereafter, the cells were collected and resuspended in 500 μL binding buffer after treatment with JS-K. Five microliters Annexin-V -FITC and 5 μL PI were then added to these cells, which were kept in the dark for 10 min. The stained cells were analyzed by flow cytometry and calculated by CellQuest software.

### Measurement of mitochondrial membrane potential

Quantitative changes of mitochondrial membrane potential(MMP)at the early stage of the cell apoptosis were measured by JC-1 probe. The cells were treated with different concentrations of JS-K for 24 h and then the cells were collected and resuspended in medium containing JC-1 reagent according to the manufacturer’s instructions. Relative fluorescence intensity was monitored by flow cytometry with excitation source at 530 nm and emissions at 585 nm.

### Nitric oxide generation detection

DAF-FM DA as a fluorescent indicator of intracellular NO was also used. The cells were plated at a final density of 5 × 10^5^ cells/well in 6-well cell plate. After treatment with JS-K for 24 h, cells were collected and resuspended with medium containing DAF-FM DA (5 μM) for 20 min at room temperature in the dark. DAF-FM fluorescence intensity was measured by fluorescence microscope and flow cytometry, respectively.

### PP2A enzymatic activity assay

The PP2A concentration was measured by Human PP2A ELISA Kit. After treatment with JS-K, the cells were collected and diluted with PBS (pH 7.2–7.4) at a density of 1 × 10^6^ /mL. Then the cells were repeated freeze−thaw cycles three times in order to release of intracellular components. The concentration of PP2A in each supernatant was measured after the cells were centrifuged at 2000–3000 rpm for 20 min and measured the optical density (OD) at 450 nm according to the manufacturer’s instructions.

### DNA transfection

Silencing of PP2A: Human PP2Ac small interfering RNA (siRNA) was used to inhibit PP2Ac, which consists of a pool of three target-specific 19 to 25 nt siRNA. PP2A-Cα siRNA (h) is a pool of 3 different siRNA duplexes(5′ → 3′): A: Sense: GCAAAUCACCAGAUACAAAtt, Antisense: UUUGUAUCUGGUGAUUUGCtt; B: Sense: GAACUUGACGAUACUCUAAtt, Antisense: UUAGAGUAUCGUCAAGUUCtt; C: Sense: GGAUAGCAGCAAACAAUCAtt, Antisense: UGAUUGUUUGCUGCUAUCCtt. The nonsilencining control as a negative control consists of a scrambled sequence. For transfection, 2 × 10^5^ cells were seeded in culture plates and transfected with PP2Ac siRNA or scrambled siRNA using Lipofectamine 3000 (Invitrogen, Carlsbad, CA, USA) for 48 h before treatment with JS-K for 24 h.

PP2A overexpression: Cells were trypsinized, collected by centrifugation, and resuspended in regular medium. A single-cell suspension was then seeded at 2 × 10^5^ cells/well (12-well plates). Cells were infected or transfected with PP2A in serum-free medium the next day. For transfection, 2.5 μg of DNA was used for each transfection using lipofectamine 3000 reagent for 4 h. Then the infection medium was removed and replaced with complete medium for 48 h. Next, cells were treated with JS-K for 24 h to observe the expressions of proteins by Western blot analysis. Negative control plasmid was treated according the above methods.

### Establishment of rat model of primary hepatic carcinoma

After one week of adaptive feeding, thirty-two male rats were randomly divided into four groups of eight each as follows: untreated control rats were received equivalent intraperitoneal injection of normal saline solution. The other rats were intraperitoneally injected with diethylnitrosamine (DEN, Sigma, USA) at 50 mg/kg body weight (bw), twice a week for four consecutive weeks. Then rats were received DEN at 50 mg/kg, once a week for another twelve consecutive weeks. JS-K treatment groups (0.25 mg/kg and 0.5 mg/kg) were given tail intravenous injection on the next day following DEN treatment, twice a week for 16 weeks. Following completion of treatment, the animals were sacrificed using an injection of chloral hydrate (400 mg/kg bw, i.p). After removing the livers, one liver fraction was frozen for further experiments, and another liver fraction was fixed in paraformaldehyde. All procedures were conducted according to our institutional guidelines for laboratory animals.

### Immunohistochemical analysis

The fixed liver tissues were harvested and fixed for 24 h. Then tissue blocks were embedded in paraffin and sectioned at 3–4 μm thickness. The slides were baked overnight in oven at 60 °C, and were then washed in xylene and hydrated in different concentrations of alcohol. Subsequently, endogenous peroxidase activity was quenched with 3% hydrogen peroxide for 20 min. To unmask the antigen, slides were submerged in citrate buffer (0.01 M, pH 6.0) at 95 °C for 20 min. After being blocked for 20 min with normal goat serum blocking solution, the sections were immunostained with the primary antibody at 4 °C overnight. The primary antisera were diluted 1:200 in PBS enriched with 0.3% Triton-X-100, 0.1% bovine serum albuminand 0.03% NaN3. Subsequently, the slides were then washed three times in PBS and visualized with 3,3-diaminobenzidine (DAB). Finally, the slides were counterstained with hematoxylin, dehydrated and mounted for imaging.

### Western blot analysis

The protein of total cell lysis, mitochondria extracts were prepared according to the manufacturer’s instructions. Protein was separated by sodium dodecyl sulfate polyacrylamide gel electrophoresis (SDS-PAGE) and transferred onto polyvinylidene difluoride membrane (PVDF, Millipore,USA). The membranes were blocked with 5% nonfat milk in TBS-T (Tris-buffered saline and 1% Tween 20) and incubated with primary antibodies at 4 °C overnight. The dilution ratio of antibody was 1:1000. HRP-conjugated goat anti-rabbit IgG or goat anti-mouse IgG were incubated for 2 h. The dilution ratio of the secondary antibody was 1:10000. Signals were visualized by Bio-Rad gel imaging and analysis system.

### Statistical analysis

All results are presented as the means ± standard deviation from triplicate experiments performed in a parallel manner unless otherwise indicated. Statistical differences were evaluated by the Student’s t-Test and considered significant at the *P < 0.05 or **P < 0.01 level. All the figures shown in this article were obtained from at least three independent experiments.

## Results

### Effects of JS-K on cell viability and apoptosis induction in HCC cells

**Fig. 1:**
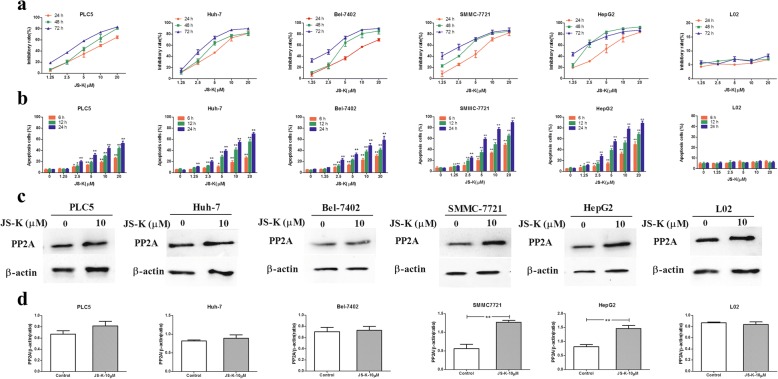
Differential effects of JS-K on cell death, apoptosis and PP2A activation in five HHC cells and L02 cells.**a** Dose-dependent effects of JS-K on cell death in the five HCC cells and L02 cells. The cells were exposed to JS-K at the indicated concentrations for 24, 48, 72 h. **b** Dose-dependent effects of JS-K on cell apoptosis in the five HCC cells and L02 cells. **c**-**d** Effects of JS-K on the expression of PP2A. The cells were treated with JS-K at the indicated concentration for 24 h. Cell lysates were prepared and assayed for PP2A by Western blotting. Data are mean ± SD. *n* = 3 for each concentration. *P < 0.05,**P < 0.01, vs. control group

The cytotoxicity of JS-K was evaluated against PLC5, Huh-7, Bel-7402, SMMC-7721 and HepG2 hepatocellular carcinoma cells using CCK-8 assay. As shown in Fig. 1a, JS-K caused a dose- and time-dependent reduction in cell viability while the cells were treated with various concentrations of JS-K (0, 1.25, 2.5, 5, 10 or 20 μM) for 24, 48 h, 72 h. The IC50 (50% inhibition of cell viability) values of JS-K for 24 h were 10.04 ± 0.22 μM (PLC5), 7.52 ± 0.70 (Huh-7), 8.23 ± 0.14 (Bel-7402), 5.77 ± 0.27(SMMC-7721) and 4.67 ± 0.67(HepG2), which indicated JS-K exerted cytotoxicity in SMMC-7721 and HepG2 cells more than in PLC5, Huh-7 and Bel-7402 cells. The tendency of cell inhibition for 48 h and 72 h was consistent with the cells treated with 24 h when the concentrations of JS-K were increasing. In contrast, JS-K at the concentration up to 20 μM did not show significant cytotoxicity against human normal hepatic cell (L02 cells).

To determine whether the cytotoxicity effects of JS-K against HCC cells resulted from apoptosis induction, Annexin V-FITC/PI staining assay was carried out. As shown in Fig. 1b, JS-K increased apoptosis rate with a dose- and time- dependent manner in SMMC-7721 and HepG2 cells more than in PLC5, Huh-7 and Bel-7402 cells. However, the numbers of cells death and apoptotic cells, especially the late stage apoptotic cells and necrotic cells, were increased after treating with indicated concentration of JS-K (10 μM) for 48 h and 72 h. The same results also observed in HCC cells after treating with indicated concentration (20 μM) of JS-K for 24 h, 48 h and 72 h. Therefore, we have selected 10 μM and 24 h of JS-K to conduct the subsequent experiments.

It was also noted that JS-K at the concentration up to 20 μM did not induce significant apoptosis in human normal hepatic cell (L02 cells). The results indicated that JS-K significantly induced apoptosis in sensitive human HCC cells, while normal human hepatic L02 cells were not sensitive to JS-K treatment.

### Effects of JS-K on the activation of PP2A in HCC cells

To clarify the role that PP2A plays in JS-K-induced apoptosis, we investigated the expression of PP2A in five human HCC cell lines through Western blot assay. As shown in Fig. 1c, JS-K caused the activation of PP2A in HCC cells, especially in SMMC-7721 and HepG2 cells. The other HCC cells (Bel-7402, PLC5 and Huh-7 cells) comparatively showed less sensitivity to JS-K- induced activation of PP2A. Meanwhile, JS-K did not significantly affect the activation of PP2A in L02 cells. These data suggested that activation of PP2A might be involved in apoptosis induced by JS-K in sensitive HCC cells. Taken together, HepG2 and SMMC-7721 cells selected as research target were related to these characteristics including higher apoptosis rate, and higher protein expression.

### Effects of JS-K on cell morphology in sensitive HCC cells

**Fig. 2:**
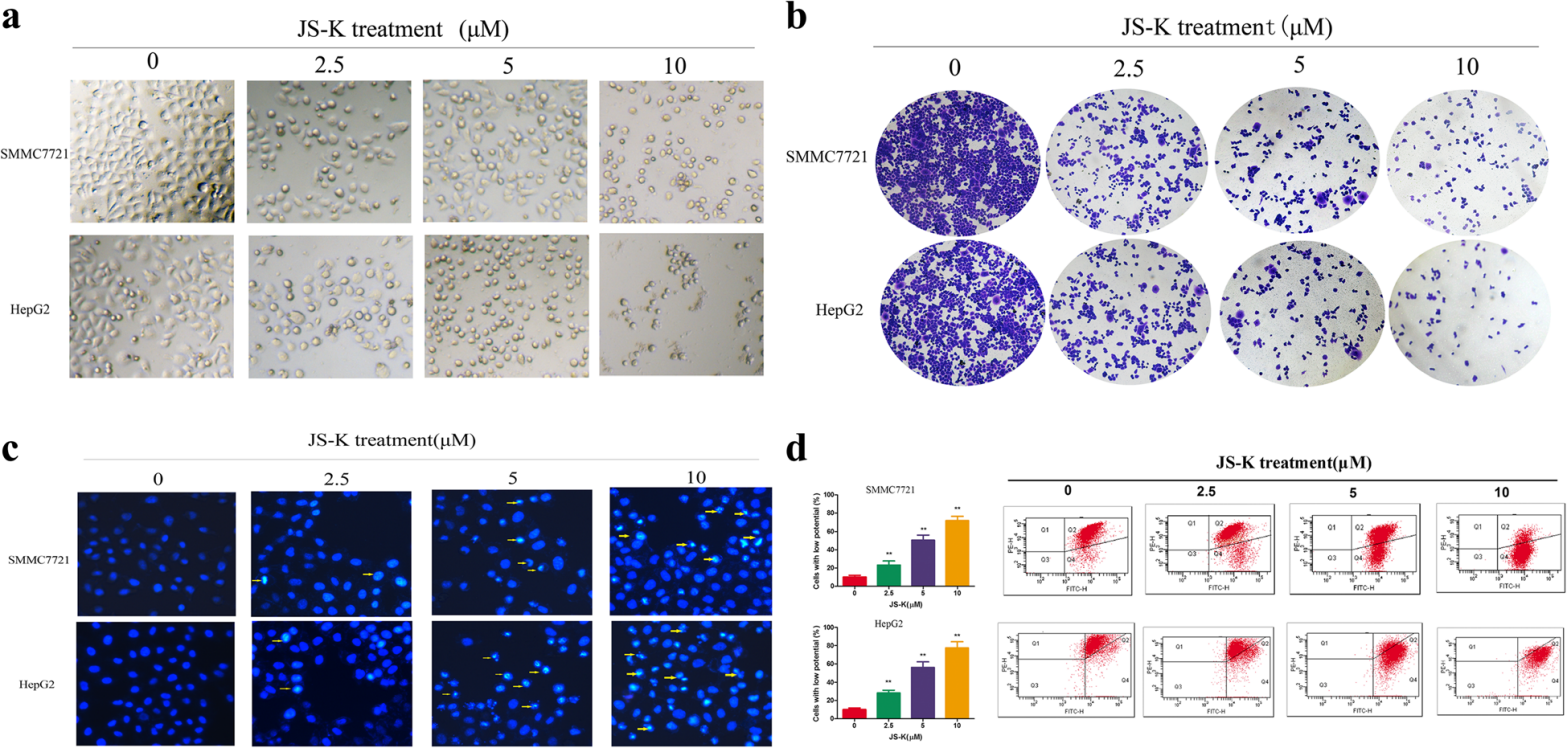
Effects of JS-K on cell morphology and MMP in SMMC7721 and HepG2 cells. The cells were treated with different concentrations of JS-K for 24 h. **a** The morphological observation were observed under a phase contrast microscopy (400 × magnification). **b** The morphological observation was determined by crystal violet staining assay (200 × magnification). **c** Morphological observation under fluorescence microscopy after Hoechst33342 staining. The arrows indicated the typical features of apoptotic cells (400 × magnification). **d** The change of MMP was detected by using JC-1 staining and analyzed by flow cytometric. The percentage in histogram of each profile represents the percentage of total cells with low fluorescence intensity. Data are mean ± SD. *n* = 3 for each concentration. *P < 0.05,**P < 0.01, vs. control group

To further validate whether the apoptosis induced by JS-K in sensitive HCC cells, we firstly observed the morphological features in JS-K-treated SMMC7721 and HepG2 cells under phase contrast microscopy. As shown in Fig. 2a, the cells treated with JS-K showed morphological changes with poor adherence, cellular shrinkage and increasing the number of floating cells, while untreated cells grew well with a clear skeletons and intact appearance. The crystal violet staining assay was further indicated JS-K could suppress proliferation in SMMC7721 and HepG2 cells except for morphological changes (Fig. 2b).To confirm the morphological changes during the process of apoptosis, the nuclear morphology of dying cells was examined using DAPI staining. As shown in Fig. 2c, the sensitive HCC cells treated with JS-K for 24 h displayed typical morphological changes of apoptosis, such as condensed chromatin and nucleus fragmentation.

### Effects of JS-K on the levels of mitochondrial membrane potential (MMP) in sensitive HCC cells

Since a loss of MMP is associated with the early apoptosis, we examined the effect of JS-K on MMP with the fluorescent probe JC-1. As shown in Fig. 2d, JS-K caused an obvious collapse of MMP both in SMMC7721 and HepG2 cells with a dose-dependent manner compared with the corresponding control group. The proportion of cells with depolarized MMP increased from (10.20 ± 1.95) % of control cells to (71.60 ± 4.75) % (10 μM) of JS-K-treated SMMC7721 cells, and from (10.17 ± 1.59) % of control cells to (77.50 ± 6.76) % (10 μM) in JS-K-treated HepG2 cells. These results suggested that JS-K-induced apoptosis was initiated by the collapse of MMP.

### Effects of JS-K on apoptosis in sensitive HCC cells

**Fig. 3:**
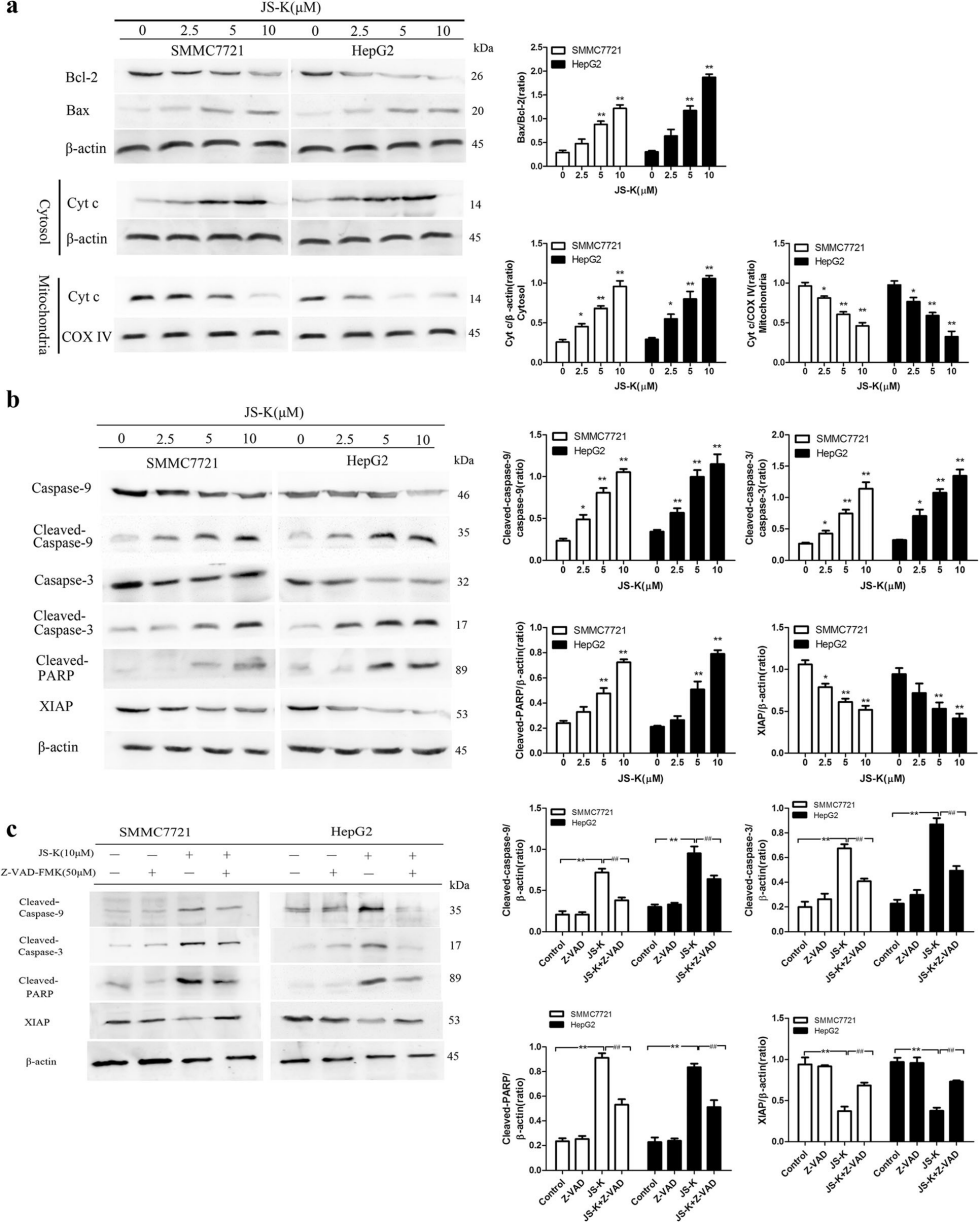
Effects of JS-K on apoptosis in SMMC7721 and HepG2 cells. **a** Western blotting analysis of Bcl-2, Bax, and Cyt c in mitochondria and cytosol of cells. **b** Western blotting analysis of caspase-9/− 3, cleaved caspase-9/− 3, PARP, cleaved PARP and XIAP of cells. **c** Effect of caspase inhibitors on JS-K-induced apoptosis. The cells were pretreated with caspase inhibitor Z-VAD-FMK (50 μM) for 1 h before treatment with 10 μM JS-K for 24 h and protein expression was assessed by Western blotting analysis. Data are mean ± SD. *n* = 3 for each concentration. *P < 0.05,**P < 0.01, vs. control group, ^#^*P* < 0.05, ^##^*P* < 0.01 vs. cells treated with JS-K alone

Moreover, cell apoptosis was also confirmed by Western blot assay. JS-K obviously triggered apoptosis in a dose-dependent manner in sensitive HCC cells. The expression of Bcl-2, Bax and cleaved-caspase-9/3 were changed. Exposure to JS-K resulted in a decrease of Bcl-2, an increase of Bax and the Bax/Bcl-2 ratio was increased in a dose-dependent manner, which is crucial for the activation of the mitochondrial apoptotic pathway. Consistently, Cyt c was released from mitochondria to cytosol in JS-K-treated cells, which implied the dysfunction of mitochondrial membrane (Fig. 3a). Meanwhile, cleaved-caspase-9/3 and proteolytic cleavage of PARP were activated significantly after JS-K treatment for 24 h in sensitive HCC cells (Fig. 3b), which provided one of the most recognizable remarks in cell death. In contrast, JS-K decreased X-linked inhibitor of apoptosis protein (XIAP) protein levels in a dose-dependent manner. These results suggested that activation of cleaved-caspase-9/3, cleavage of PARP and inhibition of XIAP ultimately contributed to JS-K-induced apoptotic process in SMMC7721 and HepG2 cells.

To confirm whether JS-K-induced apoptosis is related to the activation of caspases, cells were pre-treated with Z-VAD-FMK (a caspase inhibitor). As shown in Fig. 3c, z-VAD-FMK significantly suppressed the activation of cleaved-caspase-9/3 and of cleaved-PARP whereas increased the expression of XIAP in JS-K-treated sensitive HCC cells. The data indicated a caspase-dependent pathway might be involved in JS-K induced apoptosis.

### Effects of JS-K on intracellular NO levels in sensitive HCC cells

**Fig. 4:**
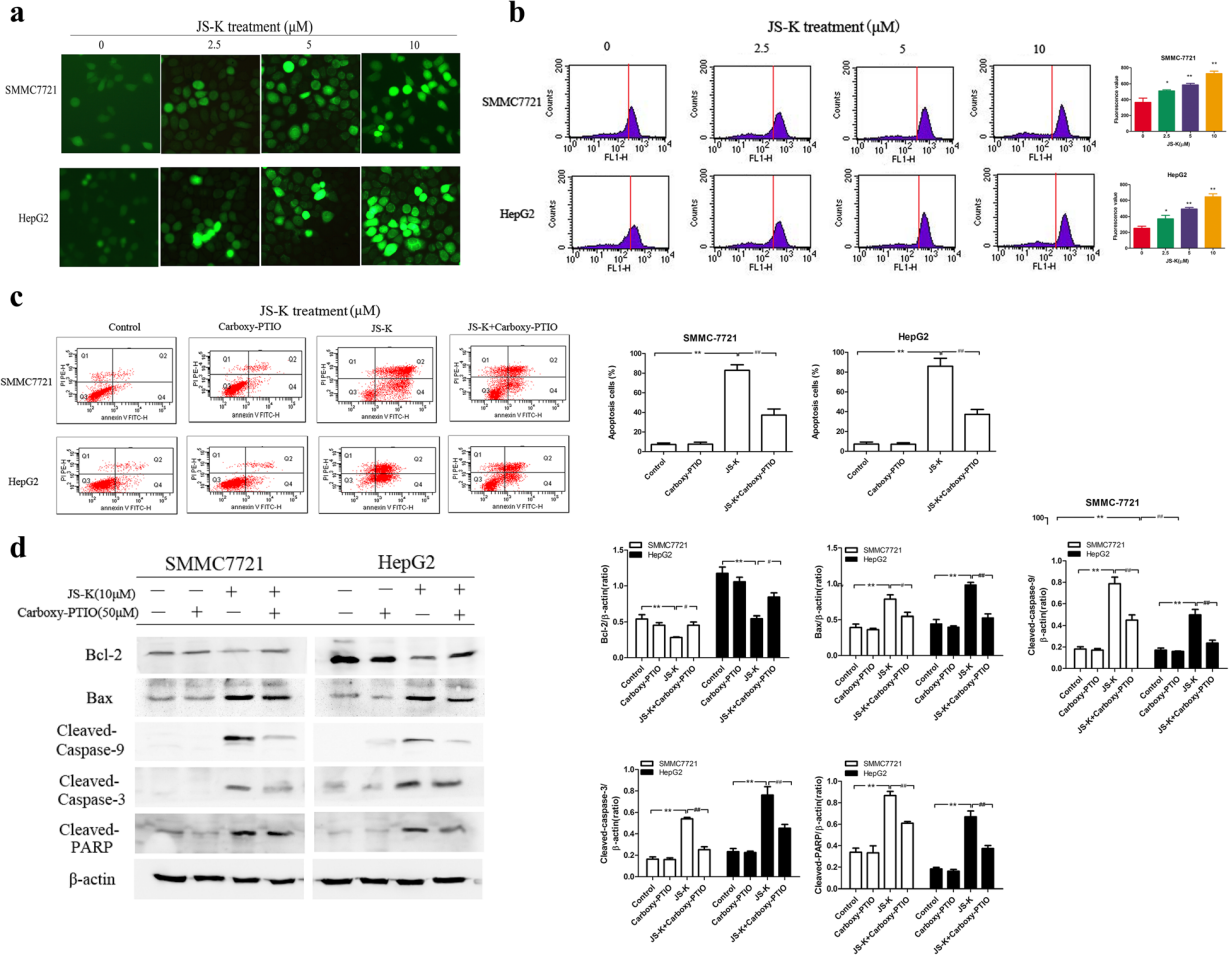
NO was involved in the apoptosis induced by JS-K. **a** The levels of NO were determined using DAF-FM DA staining by fluorescence microscope. **b** The levels of NO were determined using DAF-FM DA staining by flow cytometry. **c** Effects of Carboxy-PTIO on the apoptosis in JS-K-treated cells. The cells were treated with the NO scavenger Carboxy-PTIO (50 μM) before treatment with 10 μM JS-K for 24 h and the apoptosis was assessed by flow cytometry. **d** Effects of Carboxy-PTIO on the apoptotic-related protein in JS-K-treated cells. The cells were treated with the NO scavenger Carboxy-PTIO (50 μM) before treatment with 10 μM JS-K for 24 h and the protein expressions of Bcl-2, Bax, cleaved-caspase-9/3 and cleaved-PARP were assessed by Western blotting analysis. Data are mean ± SD. *n* = 3 for each concentration. *P < 0.05,**P < 0.01, vs. control group, ^#^*P* < 0.05, ^##^*P* < 0.01 vs. cells treated with JS-K alone

To determine whether the NO release was involved in JS-K-induced apoptosis, the sensitive HCC cells were stained with DAF-FM DA using fluorescence microscope and flow cytometry, respectively. As shown in Fig. 4a, JS-K caused a marked increase of intracellular NO levels with fluorescence enhancement whereas the untreated cells displayed low-density fluorescence. The flow cytometry assay also had the same result. As shown in Fig. 4b, the treatment with JS-K caused an increase of DCF fluorescence intensity compared to that of untreated control as the fluorescence peaks were clearly shifted to the right.

### Effects of Carboxy-PTIO on JS-K-induced cell apoptosis in sensitive HCC cells

Carboxy-PTIO was used to further validate the role of NO in JS-K induced apoptosis. As shown in Fig. 4c, Carboxy-PTIO as a well-known NO scavenger obviously reduced the apoptotic rate, ranging from (82.89 ± 5.68) % (JS-K) to (37.23 ± 6.19) % (JS-K + Carboxy-PTIO) in SMMC7721 cells and from (85.93 ± 8.05) % (JS-K) to (37.27 ± 4.94) % (JS-K + Carboxy-PTIO) in HepG2 cells. Moreover, pre-treatment with Carboxy-PTIO attenuated the JS-K-induced down-regulation of Bcl-2, up-regulation of Bax, cleaved-caspase-9/3 and cleaved-PARP activation (Fig. 4d). These results demonstrated that the effects of apoptosis induction triggered by JS-K were due to the generation of NO.

### Effects of JS-K on the expression of PP2A in sensitive HCC cells

**Fig. 5:**
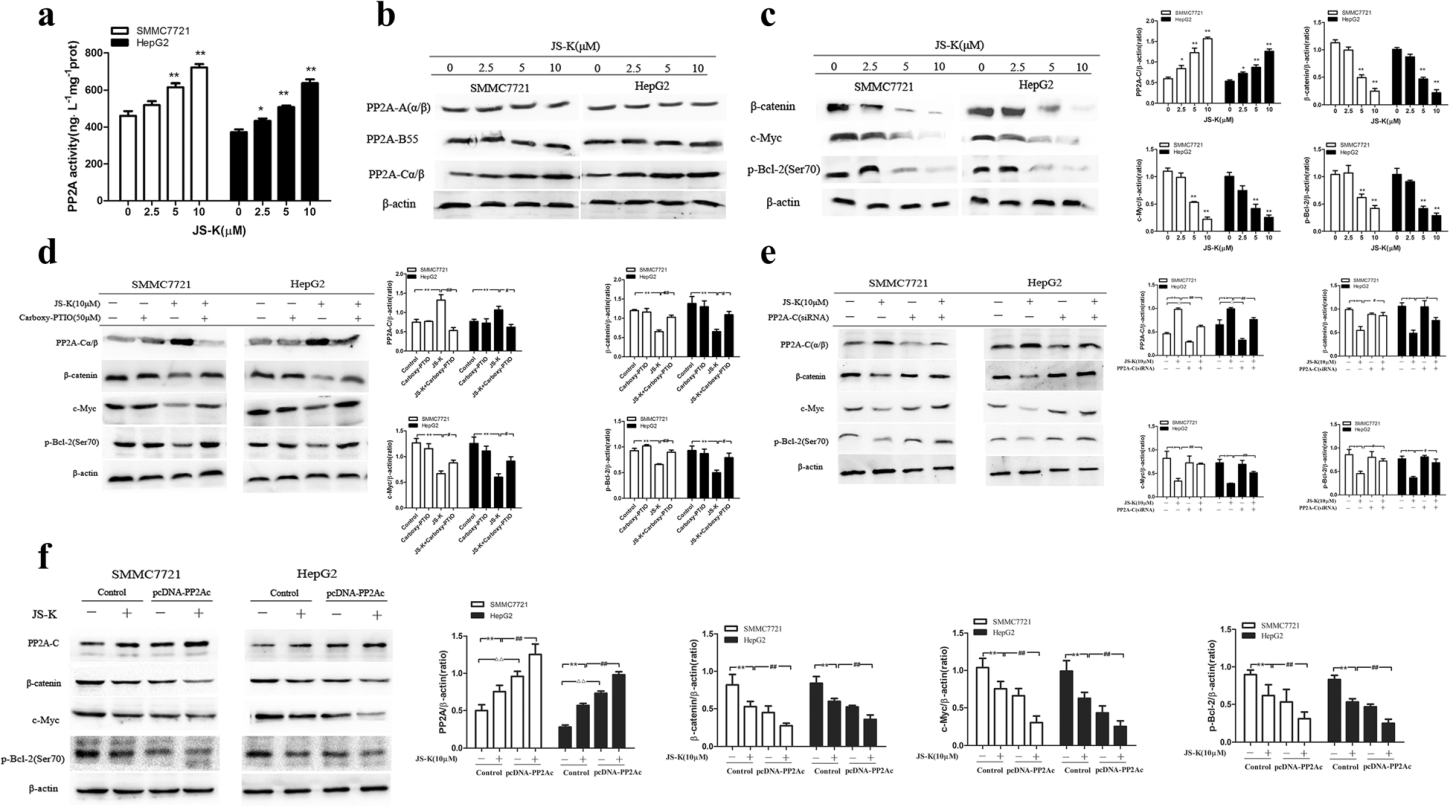
JS-K increased the activity of PP2A in SMMC7721 and HepG2 cells. **a** The levels of PP2A were measured by ELISA kit. The cells were treated with different concentrations of JS-K for 24 h and the cell lysates were prepared and assayed by enzyme-linked immunosorbent assay. **b** The effects of JS-K on protein levels of PP2A complexs. **c** Effects of JS-K on protein levels of PP2A substrates. **d** Effects of Carboxy-PTIO on the expression of PP2A-C and PP2A substrates. **e** Effects of JS-K on protein levels of PP2A substrates through silencing PP2A-C. **f** Effects of JS-K on protein levels of PP2A substrates through overexpression of PP2A-C. The cells were transfected for 48 h before treatment with JS-K for 24 h. Data are mean ± SD. *n* = 3 for each concentration. *P < 0.05, **P < 0.01, vs. cells untreated with JS-K of control siRNA /negative plasmid group, ^#^*P* < 0.05, ^##^*P* < 0.01 vs cells treated with JS-K of control siRNA /negative plasmid group, ^△^*P* < 0.05, ^△△^*P* < 0.01, vs cells untreated with JS-K of PP2A siRNA/PP2A cDNA group

ELISA assay was carried out to validate the role of PP2A in JS-K-induced apoptosis. As shown in Fig. 5a, the activity of PP2A was significantly enhanced following JS-K treatment. Notably, the treatment of JS-K could obviously change the expression of PP2A-C(α/β), while other PP2A subunits, for example, PP2A-A(α/β) and PP2A-B55, were not altered (Fig. 5b). Simultaneously, JS-K could alter the expression of the substrates of PP2A, such as β-catenin, c-Myc and p-Bcl-2 (Ser70). As shown in Fig. 5c, JS-K caused a dose-dependent decrease of β-catenin in sensitive HCC cells that was not observed in the untreated cells. Moreover, total c-Myc protein levels and p-Bcl-2 (Ser70) were also decreased in cells treated with JS-K compared to the untreated cells. Furthermore, the role of NO in mediating the expression of PP2A-C and PP2A substrates were further validated with Carboxy-PTIO in JS-K treated cells. As shown in Fig. 5d, Carboxy-PTIO could abolish the activation of PP2A-C in JS-K-treated cells. In both cell lines, pre-treatment with 50 μM Carboxy-PTIO returned PP2A substrates to near basal levels. The data suggested PP2A activation came from the release of NO. To test whether PP2A is essential for the protein changes of β-catenin, c-Myc and p-Bcl-2 (Ser70), both cell lines were transfected with PP2A/C siRNA.

As shown in Fig. 5e, the expression of PP2A protein in both cell lines transfected with PP2A/C siRNA specifically was down-regulated. Importantly, the effects of down-regulation of β-catenin, c-Myc and p-Bcl-2 (Ser70) caused by JS-K were cancelled in PP2A/C siRNA cells, but no obvious change was observed in control siRNA cells. These findings suggested that JS-K induced both cell lines apoptosis in a PP2A-dependent manner. Conversely, overexpression of PP2A could strengthen the effects of down-regulation of β-catenin, c-Myc and p-Bcl-2 (Ser70) caused by JS-K (Fig. 5f). The results also suggested that JS-K induced cell apoptosis through PP2A activation and the substrates of PP2A dephosphorylation.

### Effects of okadaic acid (OA) and FTY720 on JS-K-induced cell apoptosis and PP2A activation in sensitive HCC cells

**Fig. 6:**
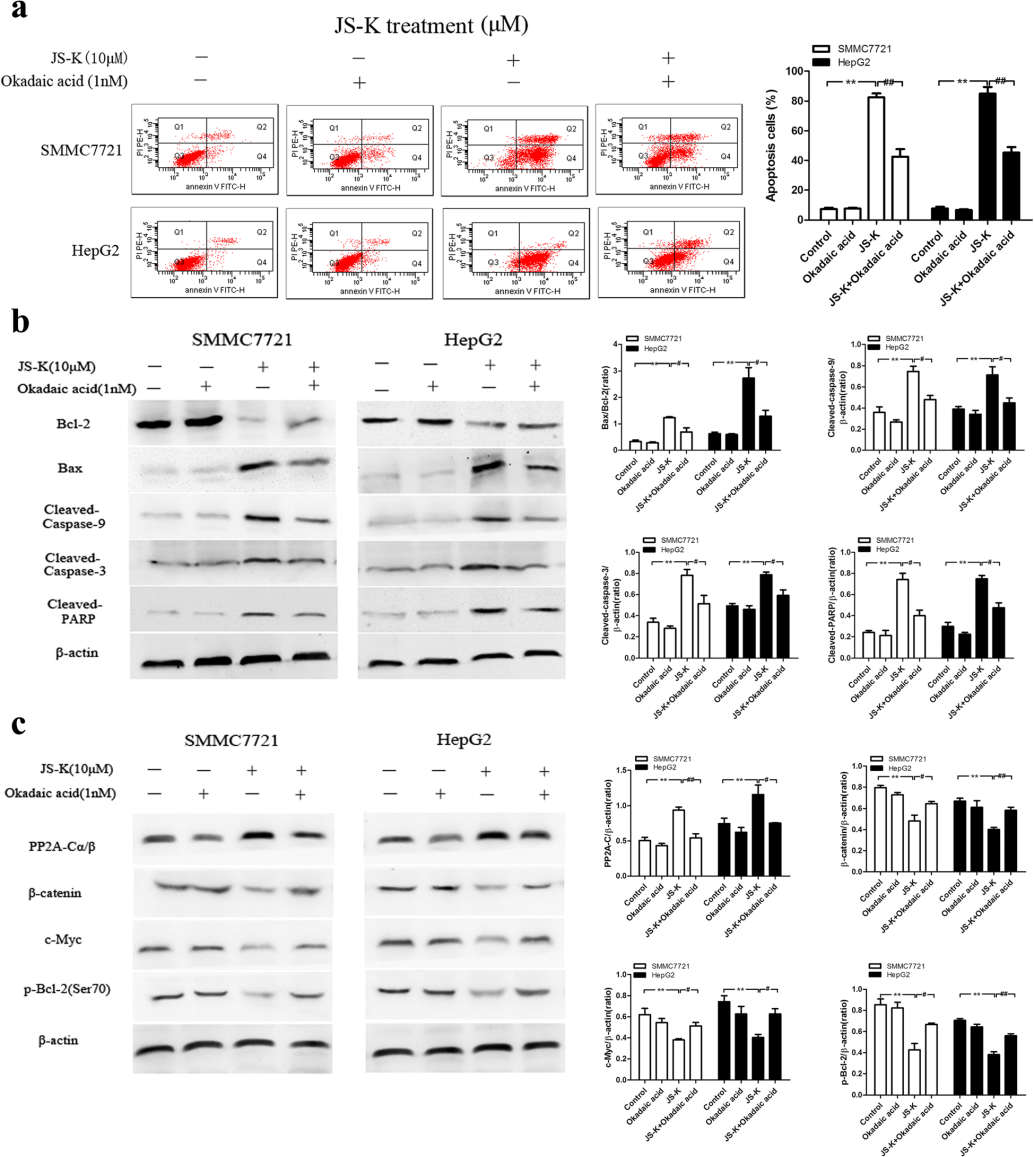
Effects of okadaic acid (OA) on JS-K-induced cell apoptosis and PP2A activation in SMMC7721 and HepG2 cells. **a** Effect of OA treatment as PP2A inhibitor on cell apoptosis. The cells were pretreated with OA (1 nM) for 1 h, and then stimulated with JS-K (10 μM) for 24 h. The apoptosis was assessed by flow cytometry. **b** Effect of OA treatment as PP2A inhibitor on the expression of apoptotic-related protein. **c** Effect of OA treatment as PP2A inhibitor on the expression of PP2A-C and its substrates. The cells were pretreated with OA (1 nM) for 1 h, and then stimulated with JS-K (10 μM) for 24 h. The expressions of protein were assessed by Western blotting analysis. Data are mean ± SD. n = 3 for each concentration. *P < 0.05,**P < 0.01, vs. control group, ^#^*P* < 0.05, ^##^*P* < 0.01 vs. cells treated with JS-K alone

To determine the roles of PP2A in JS-K-induced cell apoptosis, both HCC cells were treated with JS-K in the presence or absence of OA (a PP2A inhibitor) and FTY720 (a PP2A activator). As shown in Fig. 6a and b, pre-treatment with OA remarkably abolished JS-K-induced sensitive cell apoptosis, down-regulation of Bcl-2, up-regulation of Bax, as well as cleaved-caspase-9/3 and cleaved-PARP activation. In addition, PP2A expression was decreased with OA in JS-K treated cells. Correspondingly, the substrates of PP2A including β-catenin, c-Myc and p-Bcl-2 (Ser70) expression in JS-K treated-cells were partly abolished with OA (Fig. 6c).

**Fig. 7:**
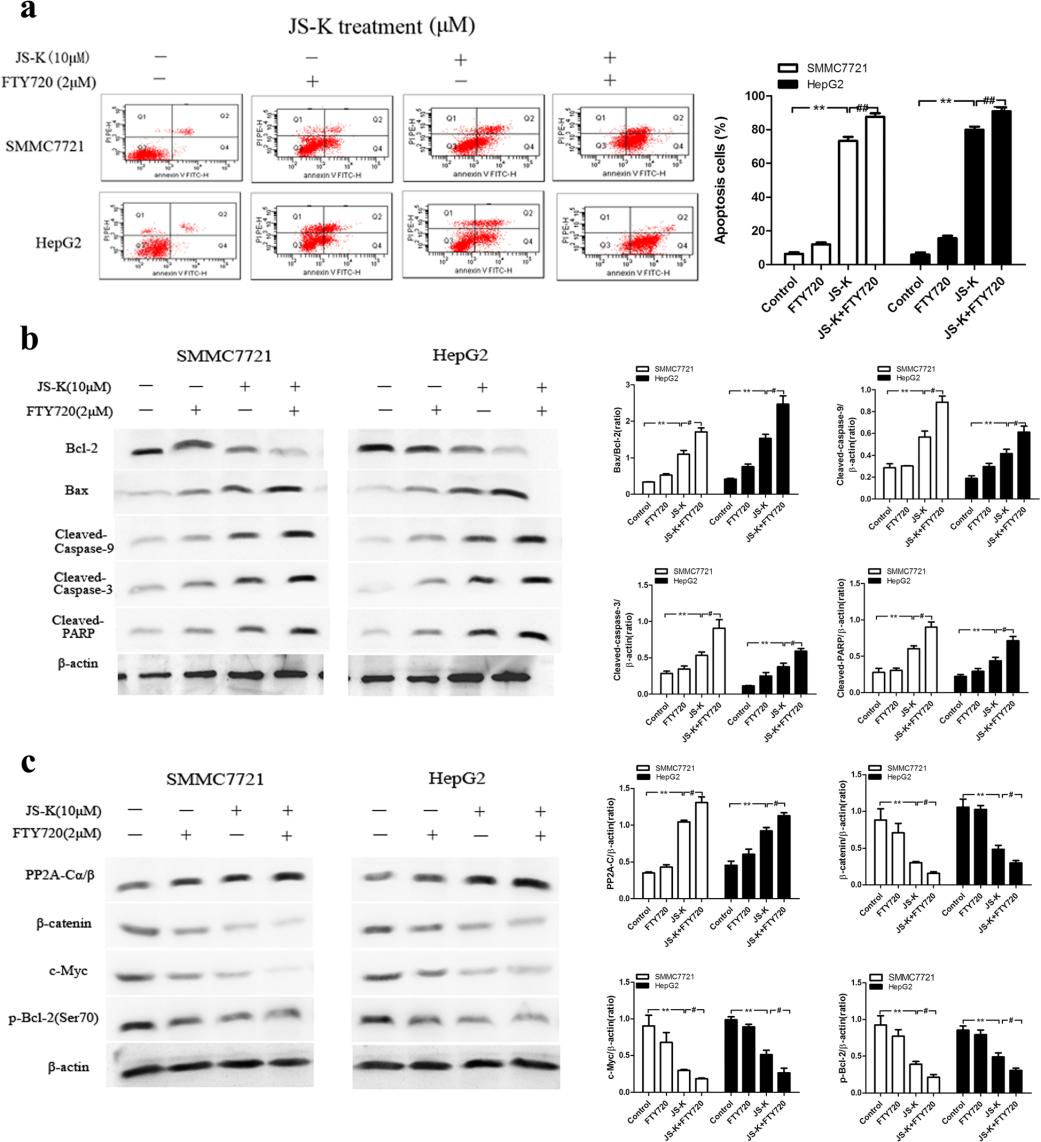
Effects of FTY720 on JS-K-induced cell apoptosis and PP2A activation in SMMC7721 and HepG2 cells. **a** Effect of FTY720 treatment as PP2A agonist on cell apoptosis. The cells were pre-treated with FTY720 (2 μM) for 1 h, and then stimulated with JS-K (10 μM) for 24 h. The apoptosis was assessed by flow cytometry. **b** Effect of FTY720 treatment as PP2A agonist on the expression of apoptotic-related protein. **c** Effect of FTY720 treatment as PP2A agonist on the expression of PP2A-C and its substrates. The cells were pretreated with FTY720 (2 μM) for 1 h, and then stimulated with JS-K (10 μM) for 24 h. The expressions of protein were assessed by Western blotting analysis. Data are mean ± SD. n = 3 for each concentration. *P < 0.05,**P < 0.01, vs. control group, ^#^*P* < 0.05, ^##^*P* < 0.01 vs. cells treated with JS-K alone

In contrast, cell apoptosis was increased with FTY720 in JS-K-treated cells (Fig. 7a). Meanwhile, FTY720 treatment enhanced JS-K-induced down-regulation of Bcl-2, up-regulation of Bax, cleaved caspase-9/3 and cleaved-PARP activation (Fig. 7b). As shown in Fig. 7c, pre-treatment with FTY720 could enhance the PP2A expression and JS-K-induced down-regulation of β-catenin, c-Myc and p-Bcl-2 (Ser70) in sensitive HCC cells. These results indicated that OA reversed JS-K-induced cell apoptosis while FTY720 exacerbated the effects of JS-K, which further verified that PP2A activation contributed to the effects of apoptosis induction in JS-K treated sensitive HCC cells.

### Effect of JS-K on the growth of rat primary hepatic carcinoma in vivo

**Fig. 8:**
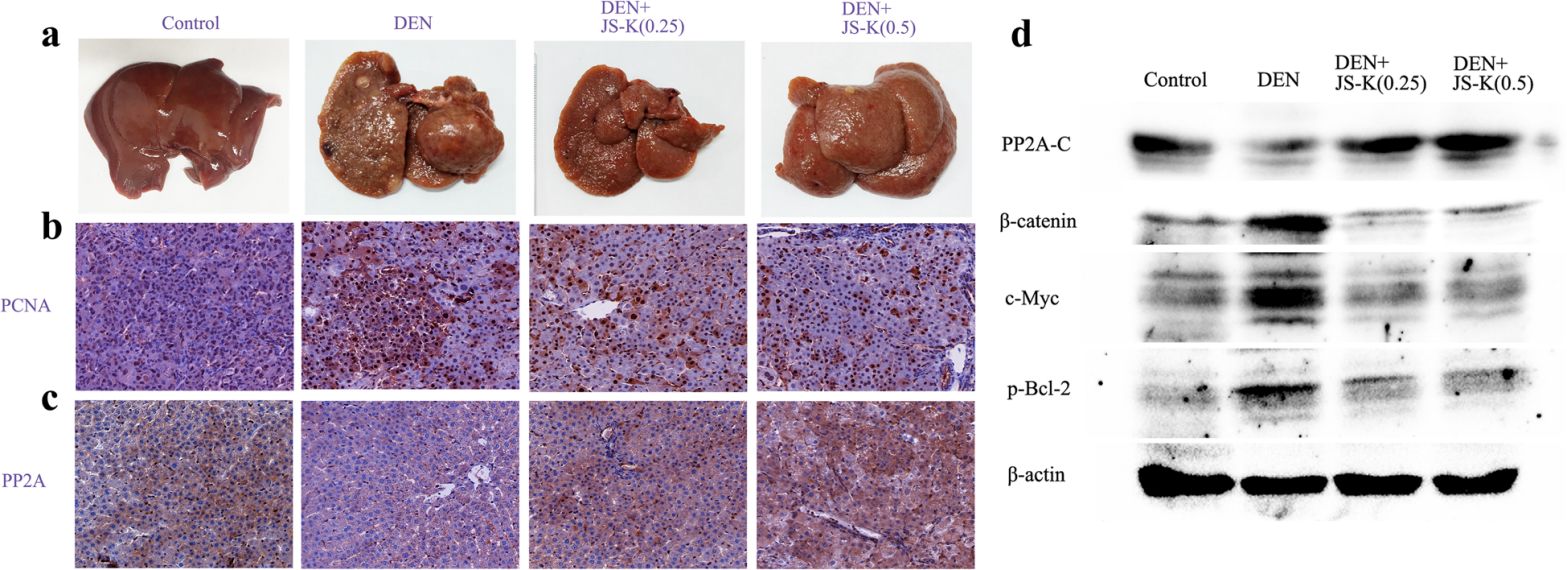
JS-K inhibits the growth of rat primary hepatic carcinoma in vivo (*n* = 8). **a** The photographs of livers in control, DEN-treated and JS-K-treated groups. **b**-**c** IHC staining of PCNA and PP2A (200 × magnifcation) in control group, DEN group, and DEN+JS-K groups (0.25 mg/kg and 0.5 mg/kg). **d** The expression levels of PP2A-C, β-catenin, c-Myc and p-Bcl-2 in rat model of primary hepatic carcinoma were analyzed by western blot

To assess gross changes in liver morphology, macroscopic lesions in liver tissue were identified in four groups. The untreated-control mice did not exhibit any evident hepatic lesions while the DEN group exhibited an irregular hepatic surface, including obvious tumor masses, several nodules and granular appearance. The liver lesions and tumor growth can be suppressed to some extent after JS-K treatment (Fig. 8a). In addition, a marked increase in the number of hepatocytes with PCNA-positive nuclei (proliferating cells) was evident in DEN group and tended to decrease with JS-K treatment (Fig. 8b). Meanwhile, JS-K-treated group showed higher expression of PP2A-C compared with DEN-group (Fig. 8c). The same results also observed in Western blot assays. JS-K treatment could induce PP2A-C activation and the substrates of PP2A inactivation such as β-catenin, c-Myc and p-Bcl-2(Ser70) (Fig. 8d). The results indicated that JS-K treatment could inhibit tumor growth possibly by activating PP2A.

## Discussion

Uncontrolled tumor cell proliferation and escape from apoptosis play the most important role in human hepatocellular carcinoma growth. Therefore, the inhibition of cancer proliferation and apoptosis induction has been regarded as the crucial target for cancer treatment. In recent years, more researchers have focused on the effects of JS-K as a novel NO donor that was involved in multiple events in cancer, including inflammation, cell cycle progression, apoptosis, migration, and invasion [13, 23, 24].

In the present study, JS-K exerted more cytotoxic effects and apoptosis induction in SMMC-7721 and HepG2 cells than PLC5, Huh-7 or Bel-7402 cells in a time- and dose-dependent manner. Moreover, JS-K hardly affected the viability of the non-tumor human hepatocyte L-02 cells. The levels of NO and the activity of PP2A were increased in SMMC-7721 and HepG2 cells following JS-K treatment, but not obviously in PLC5, Huh-7 or Bel-7402 cells, indicating that PP2A activation may be used as a novel anti-cancer target in JS-K-treated HCC cells. Thus, SMMC-7721 and HepG2 cells were selected as targeted cells in the subsequent experiment.

Apoptosis induction is a major means to eliminate cancer cells. The collapse of MMP is a typical apoptotic phenomenon in the functional impairment of mitochondria, which also facilitates the activation of caspases by regulating Bcl-2 family members on mitochondrial membrane [25]. The members of Bcl-2 family proteins are composed of both pro- and anti-apoptotic proteins. The balance between pro- and anti-apoptotic proteins groups plays a pivotal role in inducing cell apoptosis and determining mitochondrial membrane integrity [26]. Once the MMP has been disrupted, the mitochondrial permeability will be changed. Also, the pro-apoptotic signals lead to the release of Cyt c from the mitochondria into the cytosol. Cyt c subsequently activates caspase-9, which successively results in the activation of caspase-3 via cleavage induction [27]. JS-K-treated sensitive HCC cells performed the typical morphological changes of apoptosis, i.e., nucleus condensation and nucleus fragmentation. Specially, JS-K significantly increased the proportion of cells with depolarized MMP, which implied the mitochondrial dysfunction as the early signs of apoptosis was involved in the sensitive HCC cells. Accordingly, JS-K also caused a significant increase of phosphatidylserine externalization in JS-K-treated cells. In addition, JS-K effectively initiated Bax/Bcl-2 modulation, Cyt c release and cleaved-caspase-9/3 activation. The expression of PARP as a downstream substrate of caspase-3 was increased whereas the expression of XIAP as an inhibitor of apoptosis was decreased. However, Z-VAD-FMK (a caspase inhibitor) treatment abolished the cleaved-caspase-9/3 activation, PARP cleavage, as well as the JS-K-induced decreases of XIAP. These results indicated that caspases successive activation is key regulators of the JS-K-induced apoptosis in both SMMC7721 cells and Bel-7402 cells. In addition, JS-K was found to cause dramatically an apparent elevation of NO with a dose-dependent manner using two different assays. Carboxy-PTIO as a NO scavenger significantly suppressed the apoptosis, reversed up-regulation of Bax, down-regulation of Bcl-2, the activation of cleaved-caspase-9/3 and PARP cleavage in the JS-K-treated cells. These results suggested that NO can be released from JS-K, which induced apoptosis through a caspase-mediated apoptotic pathway. Protein phosphorylation is regulated by a dynamic equilibrium between the protein kinases and phosphatases. Protein phosphatase 2A(PP2A), a serine/threonine phosphatase, regulates a variety of cellular processes, including cell proliferation, apoptosis and signal transduction [28]. Accumulating evidence has been proven that PP2A as a tumor suppressor could reverse the actions of protein kinases by dephosphorylating PP2A substrates β-catenin, c-Myc, and Bcl-2 from threonine and serine residues of proteins [29, 30]. Furthermore, PP2A is genetically inactivated in many types of cancer, including HCC cells, which is composed of a scaffolding A subunit, a variable regulatory B subunit, and a catalytic C subunit [31]. The results revealed that PP2A-A and PP2A-B55 protein levels were unaffected by JS-K, but the levels of PP2A-C protein were raised in a dose-dependent manner. Simultaneously, incubation of sensitive HCC cells with JS-K caused to dephosphorylate the PP2A substrates, for example, β-catenin, c-Myc, and p-Bcl-2(Ser70).

The expression of β-catenin and c-Myc in the cytoplasm were significantly decreased, indicating the involvement of the Wnt/β-catenin signaling pathway. JS-K could modulate the Wnt/β-catenin/TCF-4 signaling pathway in Jurkat T-acute lymphoblastic leukemia cells [13]. Abnormal activation of the Wnt/β-catenin signaling pathway facilitates human malignancies. After accumulation of β-catenin in the nucleus, it binds to the T-cell factor/lymphocyte enhancer factor (Tcf/lef) family of transcription factors activates its downstream responsive substrates such as c-Myc, cyclin D1 and survivin [32]. These are related to the cell proliferation, apoptosis, cycle arrest and tumor metastasis in many human malignancies. c-Myc is induced on growth factor stimulation in normal cells and constitutively high in transformed cells. The c-Myc over expression is estimated to occur in 70% of human tumors. PP2A-mediated dephosphorylation c-Myc residue Ser62 enhances its degradation [28]. It is also found that Bcl-2 is frequently overexpressed in human malignancies, and selective phosphorylation at Ser70 could enhance its anti-apoptotic activity. PP2A is a direct negative regulator of Bcl-2 via dephosphoralation of Bcl-2 at Ser 70 (p-S70-Bcl-2) [33]. In support of PP2A acting as a activator, the results showed that knockdown of PP2A catalytic subunits impaired the PP2A-induced dephosphorylation of its downstream responsive substrates like Bcl-2 at Ser 70 whereas overexpression of PP2A has the opposite results, which demonstrated that PP2A activation was involved in JS-K-induced apoptosis of sensitive HCC cells. The addition of Carboxy-PTIO significantly abolished JS-K-induced PP2A-C activation and the substrates of PP2A inactivation such as β-catenin, c-Myc and p-Bcl-2(Ser70). Taking together, these results indicated that NO release from JS-K was related to the activation of PP2A that caused dephosphorylation of its substrates. Moreover, the anti-tumor effects of JS-K on rat model of primary hepatic carcinoma in vivo were further verified to be related to the activation of PP2A.

Notably, addition of okadaic acid (OA), a PP2A inhibitor, not only significantly abolished the effects of JS-K on the PP2A-C activation and the substrates of PP2A dephosphorylation, but also obviously protected against JS-K-induced apoptosis, such as Bax/Bcl-2 modulation and caspase successively activation in sensitive HCC cells. However, FTY720, a PP2A agonist, promoted JS-K-induced PP2A-C activation and strengthened the dephosphorylation of PP2A substrates, which further facilitated Bax/Bcl-2 modulation, cleaved caspase-9/3 activation and PARP cleavage resulting in the induction of apoptosis. These results probably implicated the involvement of the relationship between NO release from JS-K and PP2A activation.

## Conclusions

In summary, JS-K as a NO donor that can significantly inhibit HCC cells proliferation through PP2A activation, which leads to dephosphorylating or inactivating β-catenin, c-Myc, and Bcl-2, thereby inducing caspase-dependent apoptosis in sensitive HCC cells. These findings may provide the development of alternative therapy targeting the PP2A signaling network.
